# Layered double hydroxide nanocomposite for drug delivery systems; bio-distribution, toxicity and drug activity enhancement

**DOI:** 10.1186/s13065-014-0047-2

**Published:** 2014-08-10

**Authors:** Aminu Umar Kura, Mohd Zobir Hussein, Sharida Fakurazi, Palanisamy Arulselvan

**Affiliations:** Laboratory of Vaccines and Immunotherapeutics, Institute of Bioscience, Selangor, Malaysia; Department of Human Anatomy, Faculty of Medicine and Health Sciences, Universiti Putra Malaysia, Selangor, Malaysia; Materials Synthesis and Characterization Laboratory, Institute of Advanced Technology, Universiti Putra Malaysia, Serdang, Selangor 43400 UPM Malaysia

**Keywords:** Layered hydroxide, Bio-distribution, Cellular uptake, Toxicity, Drug activity enhancement

## Abstract

The production of layered double hydroxide(LDH) nanocomposite as an alternative drug delivery system against various ailments is on the increase. Their toxicity potential is usually dose and time dependent with particle sizes, shapes and surface charge playing some role both in the *in vitro* and *in vivo* studies. The reticular endothelial system of especially the liver and spleen were shown to sequestrate most of these nanocomposite, especially those with sizes greater than 50 nm. The intracellular drug delivery by these particles is mainly via endocytotic pathways aided by the surface charges in most cases. However, structural modification of these nanocomposite via coating using different types of material may lower the toxicity where present. More importantly, the coating may serve as targeting ligand hence, directing drug distribution and leading to proper drug delivery to specific area of need; it equally decreases the unwanted nanocomposite accumulation in especially the liver and spleen. These nanocomposite have the advantage of wider bio-distribution irrespective of route of administration, excellent targeted delivery potential with ease of synthetic modification including coating.

## Introduction

Layered double hydroxide (LDH) nanocomposite is a class of inorganic material with chemical composition represented by the general formula, [M^2+^_1-x_M^3+^_x_ (OH) _2_] ^x+^ [A^n-^] _x/n_⋅mH_2_O] where M^2+^ and M^3+^ are divalent and trivalent metal cations respectively [1]. The unique structure of LDH consisting of an outer positively charged metal hydroxide sheets and inner interlayer anions hydrated with water molecules aiding in its uptake and cellular penetration [1,2]. A controllable anion exchange that is pH dependent is possible due to the fascinating structure of LDH, which is also the basis of controlled-release properties of this carrier, making it a valuable choice for biological and pharmaceutical applications [3]. The synthesis and study of LDH have been on-going for decades [4]. In recent years, the synthetic production is on the increase due to their potential in electronics, drug/gene delivery, vaccine delivery system, diagnostic imaging among others [5]. In the field of biomedical application, different types of LDH were exploited for drug delivery, although they are still in the preliminary stage, but the results are promising and likely to join other delivery systems, that have reached an advanced level of clinical trials and use [6]. Some particularly interesting application of nanoparticles in general and LDH in particular includes alternative drug delivery system. This, resulted from their; local sustained release; high intrinsic pharmacological activity compared with conventional drugs. They also have improved the delivery of poorly water-soluble drugs, targeted delivery of drugs in a cell- or tissue-specific manner, transcytosis of drugs across tight epithelial and endothelial barriers (blood brain barrier inclusive), and delivery of large macromolecule drugs to intracellular sites of action [7-10]. Thus, the synthesis of LDH for drug delivery covered wide group of drugs, including but not limited to CNS diseases [7], cancers [8], anti-inflammatory and antibiotics [9] and imaging agents [10].

Below is a table (Table 1) that summarizes different type of layered double hydroxide nanocomposite synthesized by co-precipitation or ion exchange method, sizes in nanometre, type of test conducted and their outcome in relation to toxicity or drug enhancement especially against cancers.

**Table 1:** **Layered hydroxide nanocomposite toxicity and bio-distribution studies**

**Nanocomposite type**	**Method of synthesis**	**Size (nm)**	**Concentration (μg/ml)**	**Assay (Test)**	**Cell type**	**Refrence**	**Remark**
1. Mg–Al–LDH	Co-precipitation/ion exchange	50-100	40	MTT	Human kidney (N)	[4]	Contain folic acid and not toxic, more than 80% of cell viable after 3days.
2. Zn–Al–LDH	Co-precipitation	_	150	MTT	Mouse fibroblast (C)	[7]	Higher fibroblast viability with LDH-levodopa treatment than pure levodopa, LDH alone no significant effect on fibroblast
3. ZLH	Direct method	_	20	MTT	Human liver cell (HepG2) (C)	[8]	Hippuric acid (HA) intercalated ZLH showed better synergy than pure HA and tamoxefen on cancer cells.
4. Mg–Al–LDH	Co-precipitation	150-200	100	MTT	Colon cell(C)	[10]	LDH coated with chitosan also not toxic at this dose on this cells
5. Mg–Al–LDH	Ion exchange	>300		Animal single dose	Whole animal	[13]	Contain captopril and not toxic to the animal exposed.
6. Mg–Al–LDH	Co-precipitation	80-90	20	MTT	Human cervical cancer (C)	[16]	Potentiate the effect of paclitaxel
7. Mg–Al–LDH	Co-precipitation	129-149	100	MTT/Trypan blue	Cortical neuron(N)	[17]	DNA loaded LDH less toxic than pristine LDH at higher dose
8. Mg–Al–LDH	Co-precipitation	_	1000	MTT	Human osteosarcoma (C)	[18]	5-fluorouraci loaded LDH show better effect than free drugs
9. Mg–Al–LDH	Co-precipitation	50,100,200,350		MTT	Fibroblast(N) and lung(c)	[20]	Potentiate the effect of anti-cancer and milder on normal cells
10. Mg–Al–LDH/Zn-Al-LDH	Co-precipitation	_	80mg/kg of ketoprofen	Magnifying lens	Mucosal surface	[21]	Ketoprofen induced gastritis was reduced with LDH intercalation
11. Mg–Al–LDH	Co-precipitation	_	5-2000mg/kg	Blood chemistry	Balb/c mice	[26]	No significant changes to clinical and biochemical parameters and no evidence of particle retention in tissues.
12. Zn-Al-LDH	Co-precipitation/ion exchange	_	1.2	MTT	Chang liver cells (N)	[29]	Lower concentration used and no effect on viability from either the carrier or loaded LDH
13. ZLH	Direct method	_	1000	Trypan blue	Chang liver cells (N)	[30]	No Significant cell viability decrease below 125μg/ml with good anti histamine release from the intercalated cetirizine.
14. Zn-Al-LDH	Direct method	150	800	MTT, GSH, ROS, NO, comet assay,	Cervical cell (Hela) (C)	[32]	Only dose above 400ug/ml causes DNA damages, hence biocompatibility possible since it has no toxic effect at lower doses base these assays.
15. Zn–Al–LDH	Co-precipitation/ion exchange	_	50	MTT	Mouse fibroblast and human lung fibroblast cells	[33]	The toxic effect of Para-amino salicylic acid on the two cells was decrease after intercalation into this LDH
16. Mg–Al–LDH	Co-precipitation	50-300	2000	Trypan blue dye exclusion	Human Embryonic Kidney cell (HEK 293T) (N)	[34]	More than 50% of cells viable at 500μg/ml. DNA transfection successful but lower than using commercial means.
17. Mg–Al–LDH	Co-precipitation/ion exchange	57-63	40	MTT	Human gastric epithelial cell (GES-1) gastric cancer cell (MKN45 and SGC-7901)	[36]	Etoposide harmful effect on normal cell significantly reduced and its anticancer effect enhanced after intercalation into LDH.
18. Mg–Al–LDH	Co-precipitation	_	50	MTT	Breast (MCF-7) (C),cervical (HeLa) (C), and fibroblast (3T3) (N)	[37]	Not toxic to all the three cell line, but enhanced the anti-cancer effect of protocatechuic acid.
19. Mg–Al–LDH	Co-precipitation	20	50	MTT	lung fibroblast cell (N)	[38]	No toxic effect against the tested cells and bacteria. Activity of the intercalated antibiotics similar to the naked one

The table summarises some of the layered hydroxide nanocomposite activity in relation to toxicity and distribution over the last few years. The majority of whom were synthesis by either co-precipitation or ion exchange method, with sizes between 50-300 nm in most of them. Cell proliferation assay using MTT is applied in large no of the studies to evaluate for their cytotoxic and or anti-cancer impact on some selected cell lines.

Several methods have been used for LDH synthesis, among which are the co-precipitation, ion exchange, urea hydrolysis, structural reconstruction and sol-gel methods [1]. Differences in method of synthesis affect not only the crystalline nature of the particles, but also the loading capacity and quantity produced [4]. Folic acid intercalated into a magnesium aluminium hybrid via Co precipitation and ion-exchange method demonstrated 19 and 17% loading of the drug respectively [4]. Manoeuvres like sonication, hydrothermal treatment and microwave irridation usage during aging demonstrated changes to the morphology of particles [1]. However, in a drug delivery system study, most researchers demonstrated good and successful intercalation of different drugs into either zinc- aluminium or magnesium aluminium based LDH using Co precipitation and or ion-exchange methods [11,12]. The emergence of Nanomedicine is of outmost importance in the health system, more so in the area of drug delivery. Over the last few decades, several of such delivery systems have been synthesized and tested, few of which have made it to commercial stage [13]. Layered double hydroxide in particular is regarded as one of those with the least toxicity among the inorganic types [14]. They are relatively easy to synthesize in the laboratory, with different controllable sizes, shapes and other related features [1,2,4]. No single parameter has yet been identified as the one being responsible for most nanoparticles toxicity, LDH inclusive [15,16], but many physico-chemical parameters have been proposed to be critical determinants in nanomaterial toxicity, among which are; crystalline structure, surface area, oxidation status, size and chemical composition of the nanoparticle [15,16]. This review aims at discussing LDH in delivery of drugs with emphasis on wider distribution potential, organized cellular uptake mechanism, drug enhancement activity especially, anti-cancers and their characteristics decrease toxicity potential in drug delivery when compared to pure drugs.

## Review

### Bio-distribution and cellular uptake

Decade’s back, passive diffusion was shown to be the main mechanism in drug transport across biological barriers, it is a key determinant in pharmacokinetics [17]. Later studies showed carrier mediated process in drug transport across the biological membrane also played major roles, adding to the existing passive mechanism [17]. The two processes are vital in drug transport to areas of interest irrespective of admission route. In the case of LDH, the positively charged outer sheet of the delivery system is attracted by a negatively charged cell membrane, enables a facile penetration of LDH into cells [5]. A study was done previously to show the impact of surface charge on LDH and the mechanism of its cellular uptake [18]. There, a fluorescence and transmission electron microscope (TEM) were applied, to demonstrate the endocytotic cell entrance of paclitaxel-LDH nanoparticles bounded with an antibody called fluorescein isothiocyanate (FITC) into cervical cancer cells (Hela) [18]. The positive charge outer layer of LDH whose zeta potential is 20.3 mV aided in the uptake and cell penetration [18]. In order to identify the specific endocytotic pathway for LDH entrance into the cells, the researchers blocked the clathrin and caveolin-mediated (endocythotic receptors) pathways using chlorpromazine and nystatin (inhibitors) respectively on a neuronal cell. With a chlorpromazine blockage, there was no vesicle formation following treatment with LDH, but no effect clearly shown with nystatin treatment [19,20]. An anti-cancer loaded LDH was shown to enter a cervical cancer cell via the above mentioned clathrin-mediated endocytosis also [21]. The study further excluded caveolae-mediated endocytosis of the methotrexate loaded LDH into the cancer cells [21]. Some accessory cellular protein studies were used to prove the inclusion of the former and exclusion of the latter mechanism [21]. Hence, LDH cellular uptake and penetration is via endocytosis and mainly through clathrin-mediated pathways.

Further influence in LDH particle uptake is the particle size and shapes, with sizes between 50-200 nm showing concentration dependent uptake, while sizes 350 nm and above not concentration dependent [5]. Both hexagonal and rod shape LDH nanocomposite entered the cells through the same endocytotic pathway; however the hexagonal shaped particle was found to be distributed within the nucleus of the cells [5]. The prettiness of such findings is to do with possible usage of LDH in gene delivery to the nucleus in gene therapy and other related gene application. These results suggested further, the promising drug delivery potential of LDH at the cellular level without necessary damaging cell structure. Further research on synthesis and applications should focus on the size and shape specific LDH production in order to improve their actual medical application especially in areas of drug delivery.

The distribution of layered double hydroxide nanocomposite is not much different from the rest of nanoparticles, reticular endothelial system (RES) takes larger amount, especially the liver, kidney, spleen and lungs [5]. The sizes of the particle play significant role in determining the particle distribution [5]. Particles less than 5 nm are easily removed by the renal clearance; sizes more than 100 nm, are mostly sequestrated by the RES of the liver, lungs, kidney and spleen [5]. Intra-peritoneal administration of LDH to mice for five days with sizes 100 – 200 nm showed a higher distribution to the liver, lung, spleen and kidney, but not brain and/or heart [22]. Both zinc and aluminium based LDH revealed good plasma distribution of non-steroidal anti-inflammatory drug (NSAID) after oral ingestion and the analgesic effectiveness is almost similar to its counterpart (pure NSAID), added with advantage of increase gastric tolerability [23]. LDH containing antibiotics as a local drug delivery system also showed promising result [24]. This was made possible due to likelihood of sustained release and low toxicity properties of this noble carrier leading to a good drug delivery to infected middle ear in the presence of prosthesis. More recently, an *in vivo* study conducted by a group of scientist showed the distribution of LDH to liver, lung and spleen following an intravenous tail vein injection [10]. This same LDH carrier showed preference to the lung when coated with Chitosan. Further increase Chitosan concentration (higher concentration) leads to accumulation in liver and avoided the lung [10]. This preferential *in vivo* bio-distribution of LDH following modification of coating substance opened a high potential for developing organ-specific drug-delivery systems using this noble carrier both in diagnosis and therapy. In general, findings of wider distribution via different routes of administration within the range of 50-250 nm are further adding to the merits of LDH in drug delivery potential. However, more needs to be done, especially, the role of different coating material and active targeting in delivering LDH to specific areas of interest.

Invasion of brain tissue by exogenous substances/pathogens (drugs and microorganism) are strongly prevented by the blood–brain barrier (BBB) [24]. The BBB serves as a gate in preventing dangerous substances from entering the central nervous system (CNS), however, in doing so other important substances, including drugs for treating CNS pathologies like Parkinson’s disease, Alzheimer, infections and tumors are equally prevented [24,25]. Amidst these difficulties some inorganic nanoparticles of iron oxide (ION) were able to reach the brain in significant concentrations in experimental animals [26]. The same type of nanoparticle (ION) showed over 80% uptake of Amyloid beta (Aβ) in the presence of intact and un-inflamed BBB in rats [27]. LDH nanocomposite of magnesium aluminium had equally exhibited good plasma distribution after oral ingestion; it was distributed to most of the body organs, including brain even though not statistically different from control tissue samples [28]. The particle size was found to be around 100 nm, and the size is likely a part that makes it possible in achieving wider distribution of most organs, including the brain without being sequestrated by the RES [5].

Drug delivery to the brain is another emerging area in Nanomedicine [27–29]. Several surfactants have been considered in coating nanoparticle for drug delivery to the brain [29]. Nevertheless, Tween-80 coated nanoparticles enhanced transport to brain than most surfactant used [29]. Nanoparticles from copper (Cu), aluminium (Al) and silver (Ag) with size range 50 to 60 nm suspended in Tween-80 and administered parentally to experimental animal showed good brain permeation [30]. These and other related findings can be considered in future studies using LDH for drug delivery to the brain. The brain delivery of different types of the nanoparticles using Tween-80 coating is considered by some scientist to be a ‘gold standard’ [29].

### Decrease toxicity and drug activity enhancement

Layered double hydroxide nanocomposite is now emerging as potential new drug delivery system due to its low toxicity and higher biocompatibility [18,31]. Some studies have shown LDH to have the same or lower toxicity than the corresponding pure drug it carries when tested on normal cell lines [18,31]. Among which was the investigation of toxicity effects on Chang cells line, a normal liver cell using trypan blue exclusion assay at various concentrations of zinc layered hydroxide (ZLH) and cetirizine nanocomposite (CETN) [32]. CETN was found to have an IC_50_ of 617 μg/mL, whereas the ZLH showed decrease cytotoxicity with IC_50_ values of 670 μg/ml [32]. Perindopril (PE), an anti-hypertensive agent showed a similar toxicity effect on Chang cells compared to the corresponding nanocomposite intercalated with PE [31]. The synthesis was done by ion exchange and co-precipitation methods [31]. The controlled, sustained and pH dependent release property of LDH is making it biocompatible to most tissues, cell and animals as a whole. As exciting as the toxicity evaluation results of most synthesized LDH are, their application especially in drug delivery may be hindered by lack of standardization of physicochemical parameters. Among the critical considerations often missing are differences in sizes, surfaces charges and particle solubility, which may lead to results misinterpretation especially where comparism are made [33]. Nanoparticle exposure to physiological fluids is another factor that may alter the physicochemical properties leading to aggregate or agglomerate formation and possible toxicity [33].

Nevertheless, the toxicity potential of many drugs were significantly reduced after intercalation into either zinc or magnesium nanocomposite. Drug efficacies including anti-cancer potential were on the increase due to intercalation in LDH nanocomposite, it was achieved alongside decreasing unwanted anti-cancer side effect on normal cells [18,34]. Most body organs, including the brain were accessible by LDH nanocomposite to deliver different types of drug, achievable, especially with those particles whose sizes are less than 250 nm [28]. Where specific area is the target for particular drug delivery coating with various agents are yielding amazing results [10].

LDH as a drug delivery system has gone beyond decreasing toxicity of parent compound, but also potentiation of the desired effect, both *in vitro* and *in vivo* model [18]. For example, Podophyllotoxin, is an agent with poor water solubility and low bioavailability. These two negative attributes have limited the success of Podophyllotoxin in cancer treatment [18]. However, the anti-cancer activity improved following intercalation onto this nanodelivery system [18]. A time-dependent decrease in cervical cancer cells (Hela) viability seen with exposure to 20 μg/ml of paclitaxel (PPT) and its corresponding LDH nanocomposite [18]. The LDH nanocomposite showed higher toxicity (anticancer) effect than pure PPT [18]. This positive out-come is due to better cell penetration of the new delivery system, controlled and sustain release ability, increase stability of the active compound while inside the carrier. LDH itself had no significant effect on the cancer cells [18]. Other cancers where LDH’s resulted in an improved anti-cancer activity are liver carcinoma cell line (HepG2) [8]. This cancer was treated with tamoxifen alone and then followed by a mixture of either hippuric acid or its nanocomposite (HAN). This was done to study the synergistic effect of hippuric acid on liver cancer. The anti-cancer effect of tamoxifen increased about 2-fold when HepG2 cells were co-treated with HAN, whereas co-treatment with pure hippuric acid did not increase the toxicity. This shows improved anti-cancer activity as a result of synergy and believed to be secondary to HAN permeation across the cell membrane much more effectively than hippuric acid alone [8]. MTT assay for cell viability also demonstrated folic-acid–LDH complex to have better ability in reducing cancer cell viability (p < 0.01) compared to folic acid alone [34].

However, one thing in common to most LDH nanocomposite is, toxicity reduction compared to the pure drugs intercalated in them. They also demonstrated a good anti-cancer effect, even better than their corresponding pure drugs. The readily controllable sizes, plus cell’s attraction potentials of the LDH particle played role in cellular distribution and uptake. They equally play vital roles in decreasing toxicity and enhancing anti-cancer effects [35]. LDH produces some tissues and cells friendlily by-products (H_2_0, Mg^2+^, Al^3+,^ Zn^2+^) under physiological conditions (pH = 7.4 or less) [35]. This can cushion the acidification tendencies in endosomes and lysosome of cells after the nanocomposite uptake [35]. Thus, toxicity secondary to high acidity will be reduced. Recently, we reported a sustained, controlled, slow and pH dependent release of levodopa from zinc aluminium nanocomposite that lasted three-six days [7]. This slow releasing property of toxic compounds from the interlayer sheet of LDH over time is also a factor in suppressing drug toxicity.

Coating LDH with either polymers or surfactants have a role to play on the toxicity, for example coating, zinc aluminium LDH with Tween-80 further decrease its toxicity on neural cells [36]. Better dispersion due to coating may be responsible for the decrease toxicity noted in that study, other “improved” physiochemical characteristics in the coated nanomaterial are likely additional reasons. Introduction of coating affect the characters of nanoparticles like their surface charges, thermal stability, crystalline morphology, cellular/tissue uptake, target and ultimately toxicity profile in both tissue and animal exposure studies [36,37]. A related metal oxide nanoparticle (iron oxide) was coated with dextran and polymer polyethylene glycol (PEG) in another study [36]. The coated nanoparticle demonstrated decrease toxicity on the tested cells compared to their corresponding uncoated nanoparticles [38].

Microorganisms, including some bacteria are part of the human system, called normal microbial flora, they usually live in the mucous membranes and on the skin. They aid in digestion, defend the body against harmful organisms and help the immune system to mature, as such they are considered medically beneficial [39]. Antibiotics or drug delivery vehicle not capable of discriminating between the beneficial and harmful microorganism may be dangerous to human. Both Gram-positive (S. aureus) and Gram-negative (E. coli) bacterial growth was not inhibited by LDH usage as drug delivery agent at 250 μg/ml [40]. However, it successfully delivered anti-microbial silver nanoparticles that inhibit the growth of microbes [40]. This inorganic nanohybrid material allows for an efficient delivery and interaction between silver nanoparticle (antibiotic) and bacteria. It provides a flat form for possible better drug delivery potential against pathogenic organism in a sustain release fashion, without necessarily harming the medically important organism in the body in future.

*In vivo* toxicity assessment of LDH was studied via a modified Spearman-Karber statistic method called the Trimmed Spearman Karber method, in which orally administered captopril; LDH and LDH-captopril in Sprague-Dawley rats were found to have LD50 of 6590 mg/kg, 7410 mg/kg and 7315 mg/kg respectively [41]. Both LDH and LDH-captopril can be considered as relatively safe since they have LD50 that lies between 5000-15,000 mg/kg after oral dosing [41,42]. Some interesting recent advances in LDH usage as drug delivery include their use as a delivery vehicle for anti-cancer in animal study [2,43]. Methotrexate was intercalated into LDH and its anti-cancer efficacy tested. The new delivery system showed improved anti-cancer efficacy than pure methotrexate, better distribution potential and much lower toxicity in the tested animals [43]. Some of the materials used in LDH were classified medically as heavy metals with possible consequences [13,44]. Zinc for example, is a trace element needed by the body as cofactor for some enzymatic reaction [45], while aluminium is used therapeutically in medicine [46]. Malicious effects may ensure from any of these elements if taken in certain quantity or taken in the presence of altered physiology, a good example of which is in aluminium toxicity in chronic renal disease patients or in the elderly [47-49]. Other elements used in LDH synthesis poses different toxicity profiles when their chemical structure changes from stable element to free radical with oxidative potentials [50,51].

## Conclusion

However, no standard size, shape or coating has been set to assess the potential of this delivery vehicle in drug delivery. Different researchers use different sizes, concentration, shapes, tissue, cells, animals and even methodology in LDH assessment. Limited studies done to assess this noble carrier for chronic toxicity. Thus difficult or even impossible to make a generalize conclusion with regard to toxicity, distribution or drug enhancement ability. Another aspect in need of extensive research in the area of LDH nanocomposite for drug delivery is the chemical transformation tendencies of the parent metal in the body leading to free radical generation. LDH had proved to be promising in the area of drug delivery, especially in the areas of cancer therapy and MRI procedure. This nanodelivery system used either an already existing drug/compound or new drug/compound making them more efficient against various disorders/diseases and or less toxic. Though, nanoparticles size play a vital role in tissue distribution, but bio-distribution potential of these noble carriers is significantly influenced by surface coating. Some coating substances act as a targeting agent with specific delivery potential overriding the limitation caused by the particle size. The toxicity profile of this delivery system can be acceptable, as modification in size, shape, coating and functionalization could be applied to manipulate the toxicity potential where present. Generally, low toxicity and higher biocompatibility are virtues associated with LDH nanomaterial, and it is now emerging as a potential new drug delivery system. Cellular uptake of these carriers is usually coordinated; receptor mediated and energy dependent processes. As an alternative drug delivery vehicle layered hydroxide nanocomposite of both zinc and magnesium nitrate containing different chemotherapeutic agent have positively enhanced anticancer activity of so many cancer cells. They were also shown to decrease the possible cytotoxic effect of the active agents on most cell lines tested. On their own they had no anticancer or antibiotic effects, but as delivery vehicles they are yielding welcomed pharmacokinetics results. Wider bio-distribution, decreases toxicity potential, enhanced efficacy and targeted delivery will be the basis for continued usage of this nanocomposite in drug delivery system in the future.

## Abbreviations

LDH: Layered double hydroxide
MRI: Magnetic resonance image
RES: Reticular endothelial system
CNS: Central nervous system
TEM: Transmission electron microscopy
NSAIDs: Nonsteroidal anti-inflammatory drugs
BBB: Blood brain barrier
CETN: Cetirizine nanocomposite
ZLH: Zinc layered hydroxide
PE: Perindopril erbumine
ION: Iron oxide nanoparticle
MTT: 3-(4,5-dimethylthiazol-2-yl) -2,5-diphenyltetrazolium bromide
PPT: Paclitaxel
HAN: Hippuric acid nanocomposite
LD_50_: Lethal dose 50
PASA: Para-amino salicylic acid
SZNs: Salicylate-zinc layered hydroxide nanohybrids
ZnO: Zinc oxide
GSH: Glutathione assay
ROS: Reactive oxygen specie
NO: Nitric oxide assay
FITC: Fluorescein isothiocyanate
